# Repairing Boolean logical models from time-series data using Answer Set Programming

**DOI:** 10.1186/s13015-019-0145-8

**Published:** 2019-03-25

**Authors:** Alexandre Lemos, Inês Lynce, Pedro T. Monteiro

**Affiliations:** INESC-ID/Instituto Superior Técnico, Universidade de Lisboa, Rua Alves Redol 9, 1000-029 Lisbon, Portugal

**Keywords:** Biological regulatory networks, Boolean functions, Model repair, (A)synchronous dynamics, Answer Set Programming

## Abstract

**Background:**

Boolean models of biological signalling-regulatory networks are increasingly used to formally describe and understand complex biological processes. These models may become inconsistent as new data become available and need to be repaired. In the past, the focus has been shed on the inference of (classes of) models given an interaction network and time-series data sets. However, repair of existing models against new data is still in its infancy, where the process is still manually performed and therefore slow and prone to errors.

**Results:**

In this work, we propose a method with an associated tool to suggest repairs over inconsistent Boolean models, based on a set of atomic repair operations. Answer Set Programming is used to encode the minimal repair problem as a combinatorial optimization problem. In particular, given an inconsistent model, the tool provides the minimal repairs that render the model capable of generating dynamics coherent with a (set of) time-series data set(s), considering either a synchronous or an asynchronous updating scheme.

**Conclusions:**

The method was validated using known biological models from different species, as well as synthetic models obtained from randomly generated networks. We discuss the method’s limitations regarding each of the updating schemes and the considered minimization algorithm.

**Electronic supplementary material:**

The online version of this article (10.1186/s13015-019-0145-8) contains supplementary material, which is available to authorized users.

## Background

Computational biology plays a crucial role in the modern understanding of biology itself [1]. In particular, modelling helps to build systematic representations of biological systems, that can be used to simulate and make predictions in silico. However, most biological models are manually defined requiring a great amount of effort by the modeller. Also, many computational models can coherently explain the same time-series data set, and consequently, different modellers are likely to reach different models given the same data.

Models are continuously updated as we gather new information about particular biological processes. This leads to a continuous reassessment of the model consistency and its possible revision to accommodate both previous and newly acquired data. Hence, it is important to reduce the difficulty of this task by providing computational tools that allow the representation of models and further to reason over them.

This manuscript focus on signalling-regulatory networks, composed by regulatory components representing the expression level of genes or the activity of their corresponding proteins. Many mathematical modelling formalisms can be considered to represent the model evolution over time, such as Petri nets [2], piecewise-linear differential equations [3], or a logical formalism [4]. In the Boolean logical formalism [5–7], nodes are represented through Boolean variables denoting biological components and edges denote regulatory interactions between components. The set of all possible component valuations defines the state space of the system, and the evolution of the level of activity of a given component is described by logical functions combining the values of the regulators of the component. Additionally, we consider that the model dynamics can be generated considering either a synchronous or asynchronous update scheme.

When modelling biological systems, there are three main problems to be considered: (i) inferring the network topology based on data [8–10]; (ii) reasoning over the properties of a model [11, 12]; and (iii) repairing a model based on new data [13]. Here, we address the latter, while considering the logical formalism using Answer Set Programming (ASP) and focusing on the Boolean case. Note that it is possible to represent a multivalued model using only Boolean variables [14]. This work proposes the use of ASP to check the consistency and repair Boolean models of signalling-regulatory networks considering multiple time-series data sets, in the context of either the synchronous or asynchronous update scheme. Also, we consider that the structure of the original network cannot be modified during the model repair.

An increasing number of references can be found in the literature with the successful application of ASP to model and reason over biological networks [12, 15–21]. In comparison with other problem solving paradigms, the ASP declarative language is easy to model and does not require the development of sophisticated algorithms.

This paper is organized as follows. The next section introduces the necessary background on logical models and the application of ASP for the revision of Boolean logical models. Afterward, the implementation of the repair method using ASP is described. “Method evaluation” section presents the obtained results, and the last section provides some conclusions and future work.

## Preliminaries

In this section, we introduce the required definitions concerning logical formalism and ASP. We then review the literature on the use of ASP for the model repair problem.

Biological models are formal representations of complex biological processes. In this work, the formal representation uses a logical regulatory graph.

### Logical regulatory graphs

A Boolean logical regulatory graph is defined by:

- a set of *n* regulatory components $G = \{g_1, \ldots , g_n \}$, where each component is associated to a Boolean variable representing the level of expression or activity of the component;
- a set of signed directed edges *E*, where $(g_i, g_j) \in E$ with $i, j \in \{1,\ldots , n\}$ denotes a regulatory activation (resp. inibition), when the associated sign is positive (resp. negative), between components $g_i$ and $g_j$, i.e., $g_i$ is a regulator of (influences) $g_j$;
- to each component $g_i$ there is an associated logical regulatory function, $K_i : B^n \rightarrow B$ where $B = \{\texttt {false}, \texttt {true}\}$, which defines its value based on the value of its regulators;
- the value of a component $g_i$ at time *t* is given by: $g_i^{t} = K_i (g_1^{t-1}, \ldots , g_n^{t-1})$. Components without regulators are denoted as *inputs* and have constant values (either true or false).

**Fig. 1:**
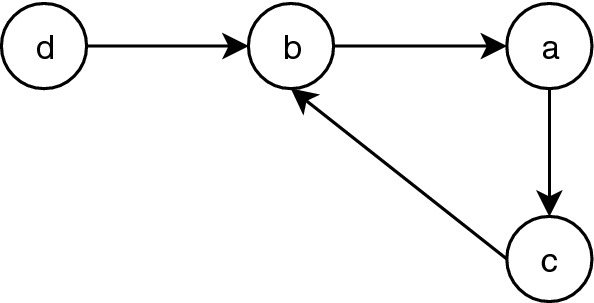
An example of a logical regulatory graphs. A logical regulatory graph with four nodes and four edges with positive sign associated

An example of a Boolean logical regulatory graph is shown in Fig. 1. The network *G* has four nodes $\{a,b,c,d\}$ and four edges with an associated positive sign.

A logical regulatory function can be defined by a combination of two basic Boolean functions (and, or), describing the evolution of a given component over time. The dynamics of signalling-regulatory networks can be represented by a state transition graph (STG) [22]. Each node, in the STG, is a state where all regulatory components have a specific expression level. The edges represent changes in the expression of one or more regulatory components.

At each time step, the set of components that may be updated simultaneously depends on the considered *updating scheme*, influencing the system evolution (see [23] for details). In the *synchronous updating scheme*, each state has at most one successor, with all components being updated at the same time. In the *asynchronous* case, each state has as many successors as the number of components called to update, exactly one component per successor [24]. Due to the associated non-determinism, it is computationally hard to generate the full asynchronous dynamics. Alternatively, a stochastic exploration can be performed by choosing randomly one successor at each time step [25]. If no component is called to be updated at a given state, then the state is denoted a *stable state*.

A *time-series data* set consists of a set of values, representing the expression level, for the elements of *G* in different time steps. Note that not all elements of *G* need to have a value in all time steps. A biological model is said to be *consistent* with the *time-series data* if and only if the value of $g_i$ at time *t* is given by $g_i^t$.

A *repair operation* is a modification to the biological model, in order to produce a new *consistent* model.

#### Boolean functions

The specification of the logical functions is typically manually performed by a modeller using any combination of the logical operators: and, or and not. To avoid obtaining different repairs for distinct, but equivalent, logical functions, a standard format to describe each function is required. In this work, we assume these functions to be encoded in Disjunctive Normal Form (DNF), i.e., a disjunction (or) of conjunctions (and) of regulators, where each regulator can be negated (not). Here, we adopt the model specification format used by *boolSim* (https://www.vital-it.ch/research/software/boolSim) [24]. The Logical Qualitative Models of biological networks library (bioLQM—https://github.com/colomoto/bioLQM) can be used to import/export models specified in different formats, including SBML-qual [26].

In general, the number of possible Boolean functions that can be used to repair a function increases exponentially with the number of regulators of the target component, following the expression $2^{2^{n}}$ where *n* is the number of arguments of the function [27]. We reduce this search space by considering only monotone non-degenerated Boolean functions. This means that each regulator always appears with the same sign (inhibition/activation) in the clauses of the function, i.e., a regulator cannot have a dual role, and that all regulators in a function play a role in changing the value of that function in at least one state of the STG.

### Answer Set Programming

In this section, a short overview of Answer Set Programming (ASP) syntax and semantics is given (for an in-depth description see [28–30]). ASP is a form of declarative programming using logical semantics [29] which has been successfully applied to model biological networks [11–13, 15, 16, 20, 21]. An ASP program is a finite set of rules and looks very similar to a Prolog program. A rule *r* has a head and a body; it is written in the following form:

$$\begin{aligned} a_{0} \leftarrow a_{1},...,a_{m}, \sim a_{m+1},..., \sim a_{n} \end{aligned}$$

where $a_{i}$ ($0 \le i \le m \le n$) is a ground atom. A literal is an atom or its (default) negation $\sim a_{i}$. The left side of $\leftarrow$ is the head of the rule and so the head of *r* is:

$$\begin{aligned} head(r)=a_{0}. \end{aligned}$$

The right side is the body, i.e. the body of the rule *r* is:

$$\begin{aligned} body(r) =\{a_{1},...,a_{m}, \sim a_{m+1},..., \sim a_{n}\}. \end{aligned}$$

The body of the rule can be decomposed as follows

$$body(r) = body(r)^+ \cup {\{~a | a \in body(r)^-\}}$$ where $$body(r)^+=\{a_{1},...,a_{m}\}$$ and $$body(r)^- =\{a_{m+1},..., a_{n}\}$$.

If the *head* of the rule is empty then *r* is called a constraint. The constraints act as filter to possible solutions. *r* is called a *fact* if $body(r) = \emptyset$. A *ground* (i.e., variable-free) instantiation of a program *P* is obtained by substituting all the variables by elements in the Herbrand universe.^1^ A (Herbrand) model is a set of (true) ground literals such that all the logical rules are satisfied (rules and default negation are considered as implications and classical negation, respectively). The solutions for a given problem, encoded using ASP, are called *answer set*s. A *model* *A* is an answer set iff *A* is the subset-minimal *model* of the *reduct*:

$$\begin{aligned} \{head(r)\leftarrow body(r)^+ \mid r \in P, body(r)^- \cap A = \emptyset \}. \end{aligned}$$

In ASP there are different types of rules that simplify the writing of a program. Examples include: cardinality constraints, choice rules, weighted rules, aggregation rules, optimization statements and conditional literals [28]. The choice rules are written as follows:

$$\begin{aligned} \{a_{0};\ldots ; a_{m}\}\leftarrow a_{m+1}, \ldots , a_{n}, \sim a_{n+1}, \ldots , \sim a_{o} \end{aligned}$$

where $0 \le m \le n \le o$. If the body is satisfied, then any subset of the atoms $a_0$ to $a_m$ can be included in the *answer sets*.

The choice rule can be bounded with at-least (lower bound) and at-most (upper bound) constraints which will be applied in the proposed implementation.

When modelling a problem into ASP, it is possible to separate the logic model from the data. The data corresponds to *facts*, specific to each instance of the problem. The logic model corresponds to the rest of the encoding which is composed of rules (called *program*). In this case, the so-called *program* encodes the properties and constraints of a consistent Boolean network and the *facts* represent the network per se (nodes, edges, functions, observed values).

In order to reason over evolving data some ASP solvers, such as *clingo* [32], provide iterative capabilities merging both grounding and solving parts of the solver. The ASP *program* is separated into three sections by the keywords: *# base*, *# step(t)* and *# check(t)*. *# base* is used to specify static rules which do not depend on the iteration step *t* (for example the observed values can be defined in this section). *# step(t)* is used to specify rules which are inferred differently depending on *t*. Finally, the rules in the section *# check(t)* are used as the stopping criterion. The iterative nature reduces the grounding problem [33], since it only grounds based on the rules/head literals of the previous iterations and not of the whole program. Therefore, the grounded program is only part of the full STG.

## Repairing inconsistent models using ASP

**Fig. 2:**
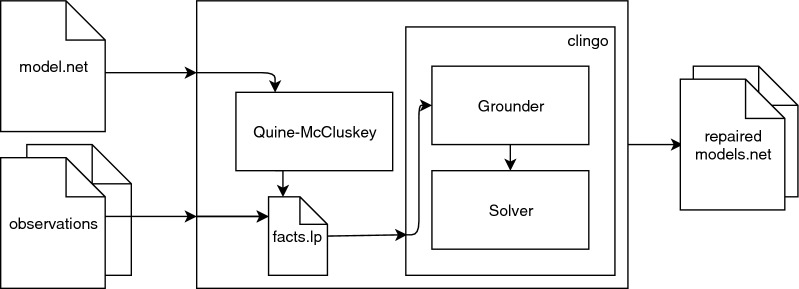
Overview of the tool. The different components of the proposed tool

In this work, we developed a tool to repair inconsistent models implemented in *C++*. The tool encapsulates an ASP solver (*clingo* [32] solver version 5.1.0) providing the user with an easy way to generate the ASP facts. Figure 2 gives an overview of the tool main components. The tool receives a model in the DNF format and one or more time-series as matrices. Not all values have to be present in the time-series matrices. If not present, the missing values will be computed according to the chosen dynamics. As the tool repairs models with different updating schemes, it is required to specify the preferred updating scheme (steady state, asynchronous or synchronous). The user can also choose which type of repairs is desirable by combining the atomic repair operations, making sure the result meets the user requirements. Finally, the modeller can also provide a list of *repairable* nodes where the problem may reside, reducing the search space and potentially the execution time. The output of the tool is all the cardinality minimal repaired models. These models are exported in DNF more precisely in the *boolSim* format. Note that, if the process is interrupted before finding the optimal solution, then the current best solution will be returned. The tool does not guarantee to return models with minimized functions since the minimization algorithm is not executed after repairing the model.

### Atomic repair operations

In this section, we describe the proposed method to correct inconsistent functions from a set of time-series data sets. We start by defining the following set of atomic repair operations:

n: Regulator negation—where a regulator can be changed from an inhibitor to an activator, and vice-versa;
s: Operator substitution—changing a Boolean operator, from and to an or, and vice-versa;
r: Regulator removal—all occurrences of a given regulator are removed from the function. To prevent the creation of components with no regulators (i.e. inputs), the removal of the last regulator is forbidden.

**Fig. 3:**
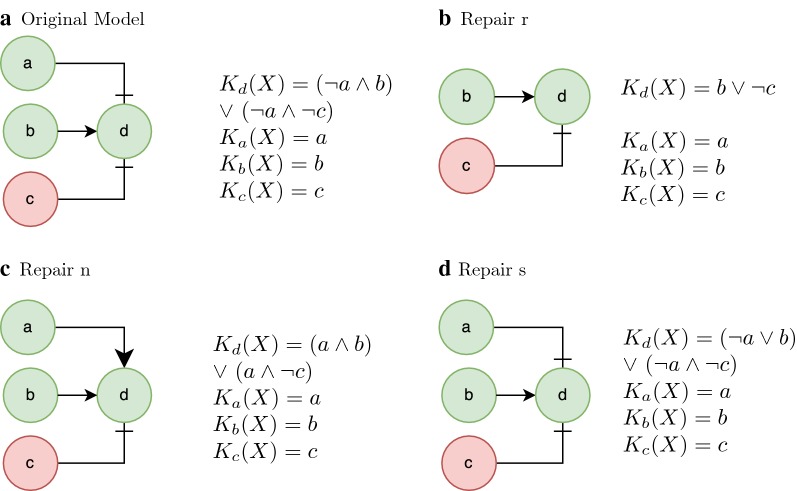
Cardinality minimal solutions for steady state. Model of a signalling-regulatory network at steady state before and after repair operations. The repair operations shown are some of the cardinality minimal solutions. Green (red) nodes represent the assignment of a node to the value true (false)

To illustrate the use of proposed atomic repair operations, let us consider a simple model and the corresponding time-series data set at a steady state, represented in Fig. 3a. This model is inconsistent with the time-series data set since the function $K_d$ cannot explain the value of component *d*. The model can be corrected by different sets of repair operations. The examples are shown in Fig. 3 correspond to different cardinality minimal solutions.

Figure 3b–d show the network and the corrected functions after applying the r, n and s repair operations, respectively.

**Fig. 4:**
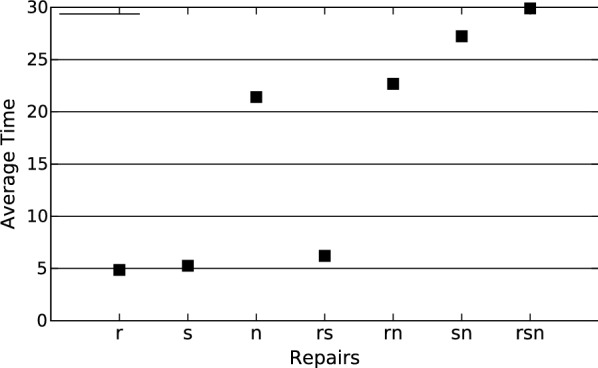
The average execution time to find the first optimal solution. Average execution time to find the first optimal solution to the networks with 10 nodes and with the number of arguments following the poison distribution with lambda 1 (and 3 time steps)

### Coverage and minimization of Boolean functions

The proposed atomic repair operations cover only a few of all possible Boolean functions. Combining repairs will allow obtaining more complex repair operations. Nevertheless, the whole space of Boolean functions is still not completely covered since these repairs depend on the structure of the function. In particular, when combining repairs of the types r, n and s for a two-argument function, a total of twelve functions are covered (all basic Boolean functions, plus one of the derived Boolean functions, the implication). Only the functions xor (exclusive or), nxor (the equivalence function), true and false are not achievable by these repairs. This is somehow expected since both xor and nxor are non-monotone functions. Table 1 shows the different combinations of repairs needed to convert the particular function $f = A \wedge B$ into a different one (whenever possible).

**Table 1:** Possible repairs for the function A $\wedge$ B and which repairs are used to achieve them

Function	Repairs used
$\lnot A \wedge \lnot B$	n
$\lnot A \wedge B$	n
$A \wedge \lnot B$	n
$A \vee B$	s
$\lnot A \vee B$	s,n
$A \vee \lnot B$	s,n
$\lnot A \vee \lnot B$	s,n
*A*	r
*B*	r
$\lnot A$	r,n
$\lnot B$	r,n
$(A \vee B) \wedge (\lnot A \vee \lnot B)$	–
$(A \wedge B) \vee (\lnot A \wedge \lnot B)$	–
true	–
false	–

Since it is possible to have different structures representing equivalent Boolean functions, we use the Quine–McCluskey algorithm [34] to obtain the prime implicants of a function.^2^ This ensures that all functions are minimized and presented in the same Disjunctive Normal Form (DNF), regardless of the initial form in which the function was expressed in. In particular, equivalent functions will share the same prime implicants and therefore share the same repairs.

Since the repair operations depend on the structure of the function, the resulting function may depend on the initial structure of the function. Additionally, the same Boolean function can be expressed in different ways, which justifies the importance of normalizing the input.

### Choosing the best repair operation

When the modeller defines a function for a given component, she has a particular network structure in mind, even if the modeller is not sure about the exact function. Here, the method searches for the cardinality minimal operation, i.e. the best repair is considered to be the one that requires fewer repair operations.

The cardinality minimal repair is not necessarily the repair that has less impact on the truth table. The consideration of the impact on the truth table would add too much overhead since it would require to enumerate the complete truth tables of all possible functions. For example, the transformation from the model in Fig. 3a into the model in Fig. 3b (removing *a* from the function $K_d$) causes a compaction of the truth table. Considering the original truth table (shown in Table 2) for the function, the output has changed in 3 lines out of 8 possible lines (the italic numbers in Table 2). Furthermore, the function can now be minimized, causing compaction of the truth table in 4 lines. This is easy to check if one knows all the values of the table. In this work, the truth tables of each function are not computed since their size grows exponentially with the number of arguments of the function. Additionally, the repair may lose the intended network structure, as shown in our toy example (from Fig. 3a to Fig. 3b).

**Table 2:** The truth table for $K_d$ before and after removing regulator a (repair *r*)

A	B	C	$K_d(X) = (\lnot a \wedge b)\ \vee (\lnot a \wedge \lnot c)$	$K_d(X) = b\ \vee \lnot c$
0	0	0	1	1
0	0	1	0	0
0	1	0	1	1
0	1	1	1	1
1	0	0	0	*1*
1	0	1	0	0
1	1	0	0	*1*
1	1	1	0	*1*

Italic values represent the changes in the truth table

### Model consistency check

The ASP implementation presented in this paper uses the incremental solving capabilities of clingo to perform an asynchronous search. Therefore, some predicates need to have an argument *t*, representing the iteration where they are inferred. The encoding described in this section repairs a Boolean network with an asynchronous updating scheme (a simpler version could be applied to steady state and synchronous updating schemes).

#### Network definition

In this section, the encoding of the Boolean logical regulatory graph is explained. Note that, the predicates explained in this section are defined in the #base section of the program. Therefore, they do not depend on *t*.

Consider Fig. 1 to illustrate the use of ASP. Each node of *G* is encoded with predicate node/1. For example, the literal node(a) represents the specific node "a", while literal node(N) is a generic representation of any node (N is a variable). A node without regulators is called an *input* node and it is represented by the predicate input/1.

The Boolean function $K_i$ associated with the node $g_i$ is represented through the combination the three basic Boolean functions. These functions can be encoded—or, and and identity—through the predicate function/2, which associates the output node of the function with the type. The type is represented by the values 0 (or), 1 (and) and 2 (identity) (e.g. function(b,1)). The output node is unique and therefore it is used to identify the arbitrary number of arguments of the function. The predicate regulator/3 associates the function with a regulator. A regulator has a sign associated (inhibition/activation) (e.g. regulator(d,b,1)).

The encoding for regulatory graph displayed in Fig. 1 is presented in Listing 1. 
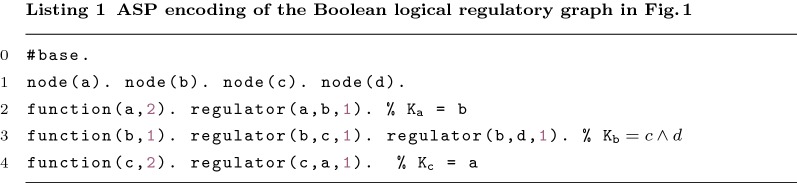


The example shown in Fig. 1 does not require the combination of functions. Nevertheless, our encoding allows it. The combination of functions is done though the definition of facts for both function and regulators (function/2, regulator/3) for all nested functions. When defining a nested function, the output may not be a node (node/1).

One may need to encode nested functions as it is shown in Fig. 3a. Function $K_d$ requires the definition of two auxiliary functions. One can encode this network using the same predicates as before. Listing 2 shows a possible encoding of function $K_d$. *abd* and *acd* represent the first two arguments of function $K_d$. These two symbols are not nodes and therefore they cannot be visited or repaired. However, they still need to be validated. 
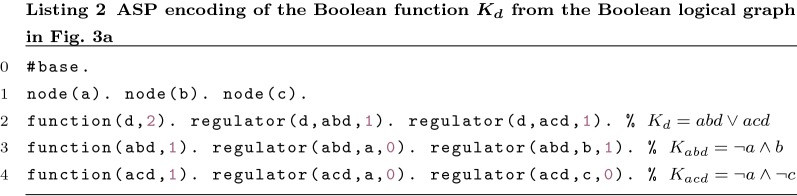


#### Time-series data

To encode each time-series data set the predicate exp/1 is used (e.g. exp($\mathtt {tS}_{\texttt {1}}$)). Predicate obs_vlabel/4 associates to each node, time step and time-series data set the corresponding observed value (e.g. obs_vlabel($\mathtt {tS}_{\texttt {1}}$,c,0,1) where 0 is the value and 1 is the time step). The predicate timeStep/1 represents the different columns of the time-series. This predicate is inferred based on obs_vlabel/4 (see Listing 4, Line 7). 
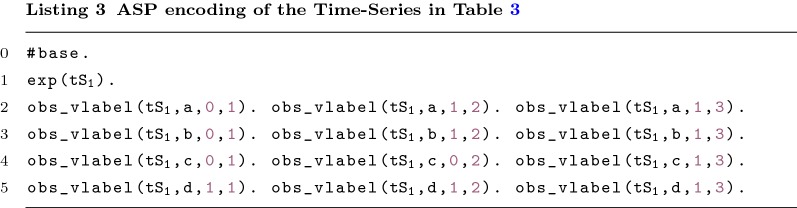


#### Asynchronous dynamics

Since the synchronous updating scheme finds little biological support, in this section we consider an asynchronous updating scheme during the repair operation procedure. We define a method to verify the consistency of the model against a time-series data set, by visiting all nodes on each time-step. As mentioned above, this method uses the iterative capabilities of clingo. The asynchronous updating scheme allows only one node to be visited at a time. Therefore, in each iteration one node is going to be visited. The search ends when all the nodes have been visited in each time step and all time steps available in the time series have been iterated (i.e. after *n* iterations, where $n= \text {number of lines} \times \text {number of columns in the time-series}$). Table 3 presents a toy time-series data set for the graph shown in Fig. 1, where the order of node visits is represented with different colours. The example is going to be executed 12 times (3 for each node). In order to visit the nodes the following rules are used: 
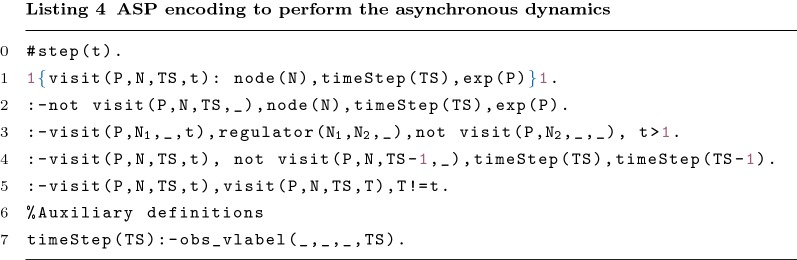

**Table 3:**
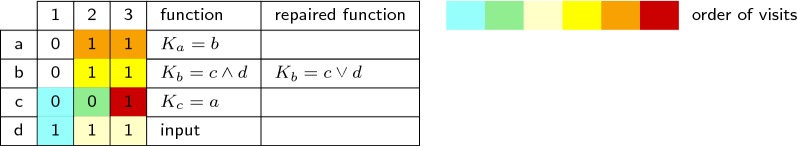
A possible order of visits by the method on a toy time-series data

On the right are the functions that needed to be repaired

The first rule of Listing 4 (Line 1) ensures that exactly one node is visited in each iteration. The four next constraints ensure that: (Line 2) all nodes must be visited in each time step present in the time-series; (Line 3) the regulators must be visited before the node they regulate; (Line 4) a node is only visited in the time step *ts* if and only if the same node has been visited in the previous time step in one of the previous iterations and (Line 5) a node can only be visited once in each time step. The constraint in Line 4 ensures the correct validation of the value on the time series given as input.

In terms of consistency checks, it is important to mention that an exception is made for the first visited node since no information about its regulators is known (it is assumed to be consistent). The search is non-deterministic and the program will choose the path that reduces the number of repairs needed (discussed further on).

This implementation allows the dynamics to be unrolled only when needed. This procedure avoids having the full state transition graph in memory.

Let us consider again the example shown in Table 3. The constraint in (Line 4) forces us to visit a node from time step 1. However, the constraint in Line 3 forces us to visit *b* before *a*, *a* before *c*, and *c* and *d* before *b*. This reduces the nodes that can be visited in the first iteration since only the first visited node is consistent by default (even without visiting its regulators). In this case, it is indifferent to visit first any of the nodes without colour. Thereupon, the rest of the nodes with time step 0 can be visited (represented in blue).

**Table 4:** Execution time, in seconds, for different models with the number of required repairs in brackets

	Arabidopsis	*C. elegans*	Budding	Fission	Mammalian
Our solution (rsn)	0.056 (1)	0.083 (4)	0.232 (8)	0.089 (3)	0.097 (6)
Merhej et al. [18]	155.224 (5)	3.369 (4)	600 (11)	20.068 (4)	600 (11)

The solution Merhej et al. uses additional rules of thumb to validate the network

Afterward, nodes *d* and *c* have the same value in different sequential time steps, the possible next steps are shown in light yellow and green. Choosing between visiting first *d* or *c* is irrelevant. However, after visiting *d* in the time step 2 one can visit the node *d* in the time step 3. In this example, we show the path requiring the fewest repair operations (see next section), and node *b* has to be visited next (yellow). Visiting *b* requires the application of repair s (changing the Boolean function). Since the value of *b* is the same as before, *b* will be visited again. Now, it is possible to visit node *a* (orange) without applying any repair operations (visiting *a* before *b* would require the application of repair operation repair n to the function of $K_a$). Finally, *c* (red) will be visited and the visiting sequence ends. For a specific visitation sequence, for the toy example, see Additional file 1: Figure S1.

**Table 5:** Prediction rate when deleting 10%, 20% and 30% of the time-series

	Percentage of errors over deleted values				
	Arabidopsis	*C. elegans*	Budding	Fission	Mammalian
10%	1	22	10	10	14
20%	0.5	12	9	17	18
30%	27	14	26	5	20

#### Consistency

The first line of Listing 5 is used to infer or not current_vlabel/3 in the first iteration of the search. current_vlabel(P,N,t) expresses that the value of *N* in the iteration *t* for *P* is 1. The Lines 3–6 are used to define the value of the visited node in this iteration. The Lines 3, 4 and 5 represent the correct propagation of the values for the functions and, or, and identity, respectively. Line 6 ensures the correct propagation of the values for an input node. Line 7 updates the current values of previously visited nodes. Lines 9–10 are used to ensure that the value is coherent with the observed value from time-series. The concept of repair/2 will be discussed further on.

Let us consider again the example shown in Table 3. The first iteration causes the inference of $visit(tS_1,b,1,1).$ This in turn could cause the inference of $current\_vlabel(tS_1,b,1)$ (Line 2). However, this would cause the constraint shown in Line 9 to be violated. Therefore, $current\_vlabel(tS_1,b,1)$ is not going to be inferred.

**Table 6:** The number of new optimal solutions found when the time-series has 10%, 20% and 30% of missing values

	Number of new optimal solutions				
	Arabidopsis	*C. elegans*	Budding	Fission	Mammalian
10%	1	3	0	0	2
20%	1	4	1	0	5
30%	2	8	1	0	5

Lines 12–15 are used to propagating the values through nested functions. The only difference to the previous lines (Lines 2–7) is the fact that they are not visited. Therefore, the propagation must happen in the same iteration and not based on the value of the previous iteration.

The value of a node must be consistent with the Boolean function associated with it. The consistency check of the network, with or without repairs, is made with the help of auxiliary predicates. The predicate oneSign/4 (Lines 19–22) indicates that a node, influenced by its associated function and based on the profile, has at least one regulator with the value true/false. The rules in the Lines 17–18 ensure that the predicates noneNegative/3 and nonePositive/3 are inferred when all the regulators of the node have the value true and false, respectively.

Above, we consider that the algorithm has already visited the node *b* in the first iteration. In the second iteration the algorithm visits node *a*. As the value of *b* is 0, it is possible to infer: $oneSign(tS_1,a,0,2)$ (Line 21). This in turn, could cause the inference of $nonePositive(tS_1,a,2)$ (Line 18).

In order to represent changes in the network the following auxiliary predicates are defined. has_function/3 represents the presence of a function for a given node. Finally, has_influence/4 represents the presence of a positive or negative influence on a node. These predicates simplify the handling of the reparations caused by the possible repair operations discussed below. 
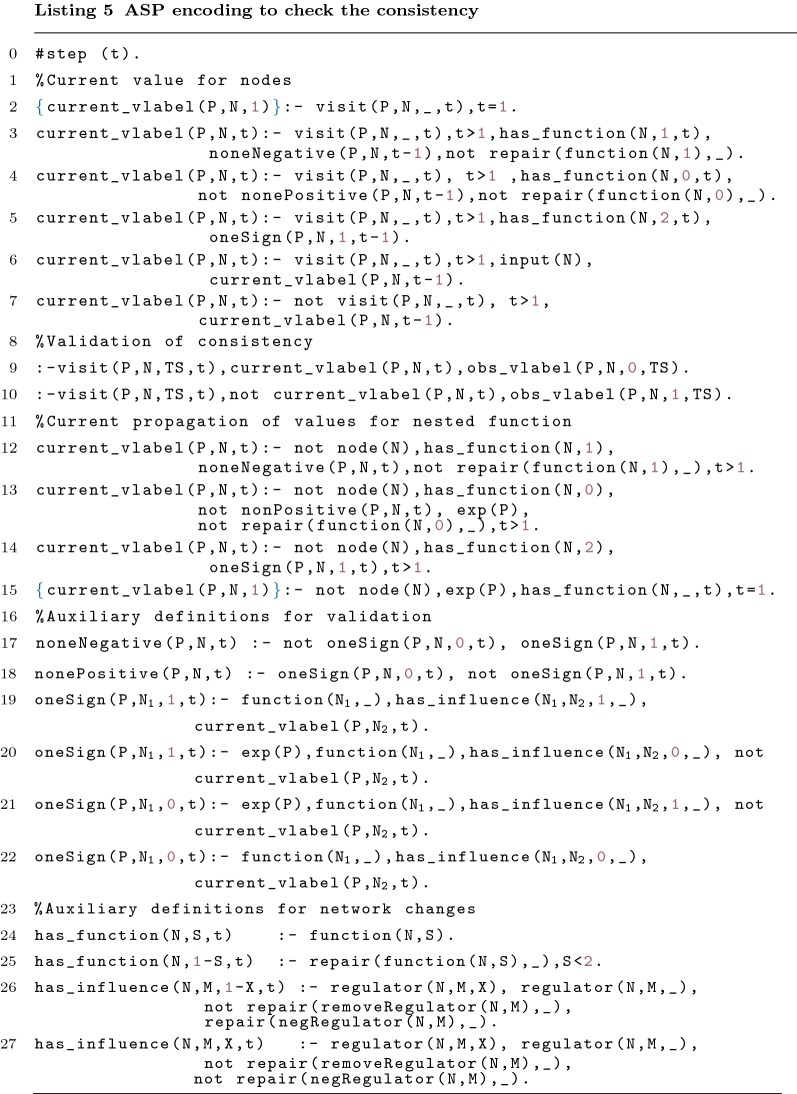


#### Repair operations

The predicate canRepair/1 indicates the nodes where the program can apply repair operations. canRepair/1 can be inferred by the predicate repairable/1, by user input, or, in its absence, the program considers all nodes as repairable (Lines 1–2). Note that these rules are only inferred at the beginning of the execution and so no information about the iteration is required.

Let us consider again the example in Table 3. In this case, it is possible to find a solution if one defines repairable(b). However, as we do not know that beforehand, all nodes have to be considered.

**Table 7:** Most common repair operation for the five networks

Arabidopsis		*C. elegans*		Budding		Fission		Mammalian	
Repair	%	Repair	%	Repair	%	Repair	%	Repair	%
reg(g7,g1)	100.00	rEdge(g2,g2)	100.00	rEdge(g11,g11)	100.00	rEdge(g1,g3)	93.33	rEdge(g4,g3)	100.00
reg(g9,g9)	61.90	reg(g6,g5)	68.75	rEdge(g4,g4)	100.00	rEdge(g6,g3)	86.67	rEdge(g4,g4)	100.00
reg(g4,g3)	57.14	rEdge(g4,g5)	62.50	rEdge(g7,g10)	100.00	rEdge(g7,g3)	86.67	rEdge(g9,g8)	100.00
reg(g10,g7)	57.14	reg(g3,g7)	62.50	rEdge(g7,g3)	100.00	rEdge(g9,g3)	83.33	rEdge(g2,g6)	98.08
reg(g7,g7)	52.38	rEdge(g5,g5)	56.25	rEdge(g7,g7)	100.00	rEdge(g9,g2)	73.33	rEdge(g2,g4)	96.15
rEdge(g2,g9)	47.62	reg(g7,g6)	56.25	rEdge(g8,g8)	100.00	rEdge(g4,g3)	70.00	rEdge(g1,g10)	94.23
reg(g6,g4)	47.62	rEdge(g5,g7)	50.00	rEdge(g1,g2)	97.30	rEdge(g6,g2)	70.00	rEdge(g5,g7)	94.23
reg(g7,g9)	47.62	funcAND(g2)	43.75	rEdge(g1,g5)	97.30			rEdge(g9,g7)	92.31
		funcAND(g5)	43.75	rEdge(g7,g9)	97.30				

*rEdge* stands for removing an edge, *reg* changing the sign of regulator, *funcAND/funcOR* changing the function

For each type of repair the predicate pos/2 is inferred if it is possible to apply the repair. Line 3 shows when it is possible to switch an or to an and function (and vice-versa). The literal $repair\_s$ represents the activation of repair s. Lines 4 and 5 show the rules to negate and remove a regulator, respectively. $repair\_n$ and $repair\_r$ represent the activation of the respective repair operations. Note that it is impossible to remove all regulators (Line 5).

The generation rule in Line 6 allows generating 0 or more repairs from the possible repairs found. The ASP solver is going to minimise the number of repair operations applied to the network, through the statement shown in Line 7.

Let us consider once again the example in Table 3. In this case, it is possible to find all types of repair operations. It is possible to remove regulator *c* or regulator *d* (but not both) from function *b*. Still relating to function *b*, it is possible to switch from an and to an or. Furthermore, it is possible to negate all four regulators. Recall that it was necessary to perform a repair operation to visit node *b* in the second time step ($visit(tS_1,b,2,8)$). The program infers *repair*(*function*(*b*, 1), 8) from the list of possible repairs. 
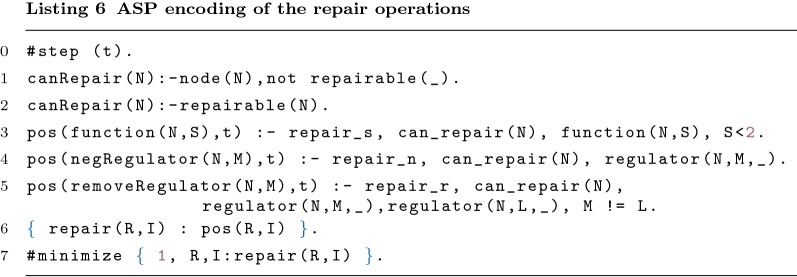

**Table 8:** Percentage of satisfiable instances and number of repairs needed to return consistency, for the five synchronous networks, considering different sizes of the repairable nodes list

	Arabidopsis	*C. elegans*	Budding	Fission	Mammalian
20%					
%Satisfiable instances	10	0	0	0	0
#Repair	1				
Repairable node list size	2	1	2	1	2
30%					
%Satisfiable instances	36	0	0	0	0
#Repair	1				
Repairable node list size	3	2	3	2	3
50%					
%Satisfiable instances	58	2	0	4	6
#Repair	1	4		3	6
Repairable node list size	5	4	5	4	5
70%					
%Satisfiable instances	72	6	2	4	24
#Repair	1	4	8	3	6
Repairable node list size	7	5	7	6	7
90%					
%Satisfiable instances	92	10	4	10	74
#Repair	1	4	8	3	6
Repairable node list size	9	7	9	8	9
Network size	10	8	11	9	10
#Inconsistent nodes	1	4	7	3	5

The first column represents the percentage of repairable nodes in relation to the network size. For each list size, there are 50 randomly generated lists. The number of inconsistent nodes in each network is also present

## Related work

Ostrowski et al. [9] successfully used ASP to infer networks based on time-series data. The objective is to find all networks that satisfy the time-series data sets. To achieve this goal, all combinations of edges and Boolean functions are tested. The considered dynamic allows any number of components to be updated at the same time. Another approach is to use genetic algorithms [35] to optimize Boolean networks from time-series data. These authors consider an asynchronous updating scheme to generate the dynamics. The training set is a set of time-series data which the model has to reproduce. Considering that the original models are large, it becomes difficult to reason over these models. With this in mind, the objective is to find the smallest possible sub-network to describe all the experimental values. However, not all nodes can be removed. These nodes are defined by the user and can represent key experimental readouts. Moreover, the optimization process tries to maintain the largest possible number of edges, removing only the edges that are inconsistent with the time-series data.

Abdallah et al. [12] implemented an ASP-based tool following the discrete formalism called the Process Hitting. The objective was to use an abstraction to model large synchronous networks in order to study their properties. This abstraction is useful when dealing with very large networks. The properties inferred with this abstraction are properties of the original network, avoiding having to test them in the original network. However, if a behaviour is impossible in the abstraction, nothing can be inferred about the real network.

Rocca et al. [21] proposed two possible routes to validate biological networks using different methods. The first method discussed uses the Boolean method to validate the consistency of the networks. The method was implemented using ASP with an explicit definition of the asynchronous dynamics. The ASP encoding proposed by Rocca et al. [21] to encode Boolean functions does not scale correctly. The encoding requires the definition of specific rules for each function with different arity. Therefore, every time a function with a different arity is required, new rules need to be added. As the solution proposed by Rocca et al. [21] uses an STG [22], it consumes an unnecessary amount of memory given that the complete dynamics is always defined. When considering this method, the authors do not propose any type of repair operations. Only when considering the Thomas method [36], the authors proposed repair operations. The latter add threshold parameters to explain the dynamics of the network. The repair operations are based on changing the predicted properties to guarantee consistency with all time-series data. The work considering the Thomas method was later extended with an ASP-based automatic tool to detect and repair inconsistencies in a biological model [19].

Recently, Merhej et al. [17, 18] successfully modelled biological networks in ASP using a synchronous updating scheme. In this work, the authors also proposed to repair a model resorting to the addition and removal of regulators, based on a set of pre-defined rules of thumb.

## Method evaluation

In this section, we evaluate and compare our method with the one recently proposed by Merhej et al. [18], the synchronous updating scheme.

The authors consider five models and their respective time-series data sets: Arabidopsis [6], Budding Yeast [37], *C. elegans* [38], Fission Yeast [39], and Mammalian [40] containing 10, 11, 8, 9 and 10 nodes, respectively. The numbers of time steps vary from 10 to 13. We chose a default function for these models where a node is active whenever there is at least one activator and no inhibitors present. This approach is similar to the activation rule proposed by Merhej et al. [18], except that, in our case, the updating constraints are more precise, since they are expressed by a Boolean function. The difference lies in the case where, at a given time step, a gene is active and there are no activators and no inhibitors. The Boolean function states that on the following time step, the gene will become inactive, and Merhej et al. activation rule states that the gene stays active, since there are no inhibitors.

The tests were executed using the *runsolver* tool [41] with a time out of 600 s and a limit of 3 Gb of memory. The implementation was run on a computer running *Ubuntu 14*, with 24 CPUs at 2.6 GHz and 64 Gb of RAM.

Since our method considers precise Boolean functions, we would expect it to be slower due to the number of possible functions considered for each model component. However, Table 4 shows that our approach is faster by at least two orders of magnitude than the approach proposed by Merhej et al. [18], with thumb rules. The solutions found by our method also have fewer repairs with respect to the original model. The method proposed by Merhej et al. considers additional constraints like the network diameter that may play a role in the running time and minimality of the solutions.

**Table 9:** Execution time (in seconds) for repairing networks with the repair *s* and lambda 1

# of nodes	Time steps				
	3	5	8	10	15
10	5.46	18.07	56.24	109.67	139.93
20	12.31	47.64	233.04	337.20	–
25	35.18	512.12	537.94	–	–
50	146.80	–	–	–	–

Next, to test the system capable of dealing with missing entries in the time-series data set, for each species (Arabidopsis, Mammalian, Fission, *C. elegans*, and Budding) we generated 10 files. From each file, values were randomly removed, following an uniform distribution. These incomplete data sets were tested using our approach with the stopping criteria of reaching an optimal solution. However, it is possible that the first optimal solution found is not the closest solution to the original data sets. With this in mind, Table 5 shows the percentage of incorrect values found when deleting 10%, 20% and 30% of the data present on the time-series. A value for a node is incorrect if it is not the same as the value in the original time series. As expected, as we increase the number of deleted values, it gets harder to correctly recover the original values. For example, in the Arabidopsis data set, the difference between the number of incorrect values when removing 10% and 20% is smaller than when removing 20% and 30%. Note that the percentages shown on Table 5 are based on the number of deleted values and not on the complete data set.

Since removing values may change the number of repairs needed, which may influence the prediction results, Table 6 shows the number of files for which there was a better solution in terms of repair operations.

When considering the *C. elegans* data sets with 30% of missing values, almost all instances found a better solution (8 out of 10). The *C. elegans* data set with a higher number of incorrect values is also the data set for which the algorithm improves better the solution, in terms of cardinality.

Also, due to the existence of different solutions given by the tool, we studied what all of them had in common. So, for each of the species, the tool was run until the first optimal solution was found, keeping also all the non-optimal solutions found previously. For each species, we compared these solutions, in order to find the most common repairs, which would represent the most essential operations to be made to the model. Keeping in mind that the results may be influenced by the search made by the ASP solver since we do not enumerate all answers, Table 7 shows the top 10 most common repairs in the solutions obtained. The knowledge of the most common repairs may act as an additional criterion, providing some clues to the modeller to choose between different repaired models.

Finally, the tool described in this document allows the user to define a list of nodes whose functions can be repaired. In order to test this feature, lists of different sizes were randomly generated. The lists contained 20%, 30%, 50%, 70% and 90% of the nodes from the model. For each of these list sizes 50 different sets of nodes were generated. Note that for lists containing 90% of the nodes the number of different combinations can be lower than the number of generated files. Since the considered updating scheme is synchronous and their time-series matrices are complete (no missing values), no propagation of values happens. For this reason, the repairs found are always the same (i.e. affect the same node). With these conditions, when it is possible to repair the network, the solution is the same as for the complete network. For all tests, the execution time was below 1 s. The percentage of satisfiable instances varies with the size of the list as one can see in Table 8. As expected, the percentage of satisfiable instances found increases when the size of the list grows. This table also shows the minimum number of inconsistent nodes which need to be in the list in order to repair the network. For example, for the Budding Yeast network the node lists with less than 7 nodes will never be able to repair the network since this network has 7 inconsistent nodes. This functionality allows the modeller to repair a network, focusing the repair only on a small part of the network.

### Asynchronous dynamics

After checking that the program was able to repair models using the synchronous updating scheme, we randomly generated instances of time-series data to evaluate the program when considering an asynchronous updating scheme. The motivation to consider an asynchronous dynamics is the fact that multiple components in the time-series data may not be acquired at the same time points. This relaxes the synchronism between components, therefore increasing the search space considerably.

#### Characterization of the data sets

The randomly generated instances were separated into different categories in order to evaluate the scalability of the proposed solution. First, the model and the respective functions were generated through a script that creates random *GINsim* models (available at https://github.com/ptgm/BoolNetR2GINsim). With this script it was possible to generate different models with different numbers of components (10, 20, 25, 50 nodes) and the arity of each function would follow Poisson distribution (with lambda parameter^3^ 1, 2 and 4). The type of the function (and, or) was randomly generated following an uniform distribution. The data sets were produced by running the implemented ASP program. Since these data sets (with different number of time steps 2, 3, 5, 10 and 15) are by default consistent with the model, we introduced some random changes in the data, considering 5% of changed values (randomly generated based on the uniform distribution).

#### Results

Tests with 100 or more nodes, even with only two-time steps and a lambda value of 1, are difficult to run within the imposed time out, since just the propagation of values for the network takes on average 500 s.

All executions that did not time out found an optimal solution without needing any repair operations, i.e. only by choosing an order of visit. As one can see in Fig. 4, repairs r and s are faster since they do not need to change the structure of the network. Negating a regulator (repair n) is slower than applying repair r since the program internally adds new edges and nodes when applying this repair, which increases the search space.

Table 9 shows the CPU time required to find an optimal solution using repair s. One can see that with a 10 component model, it is relatively fast to obtain a solution even for a large number of time steps. Expectedly, the growth in the number of components is accompanied by an increase in the execution time. For example, it is impossible to repair the network within the time limit when considering 50 components and 5 time steps. With more than 50 components, the search space makes it even harder to repair a model within the time limit.

The overhead introduced by the Quine–McCluskey minimization algorithm is mostly constant throughout the different tests. However, when one looks at it from the point of view of the percentage of time spent by the tool it can be seen that it depends on the size of the network. For the tests with two-time steps and with 10 nodes, this percentage is around 15%, while with the tests of 50 nodes (still with two-time steps) the percentage is around 1%. Moreover, the weight of the minimization algorithm decreases when the number of time steps increases, since the program spends more time solving the network with functions having the same level of complexity. So, the minimization algorithm adds little overhead for normal size networks, which is a good price to pay for having a normalized input with minimal functions.

## Conclusions and future work

In this work, we proposed an ASP-based tool capable of repairing the logical functions of a Boolean logical model, in order to make it consistent with a (set of) time-series data sets. The extension to multivalued logical models would be straightforward by applying a Boolean mapping [14].

The proposed tool considers a specific input and output (*boolSim* format), which can be obtained from SBML-qual [26] or other formats through the bioLQM library (https://github.com/colomoto/bioLQM).

The tool was able to find an optimal solution for all considered models, showing a significant increase in performance when compared to the ASP encoding proposed by Merhej et al. [18].

We also created data sets for all time-series with increasing percentages of missing values. We show that the method is robust, being capable of verifying the model consistency and retrieving a repaired model even with 30% of missing values. We could also retrieve the most common repair operations, thus providing the modeller with additional information to choose among the retrieved solutions.

Regarding the extension for an asynchronous search, we show that the running time is still acceptable considering the current model sizes. However, one could limit the asynchrony between components by adding a sliding window of size *k*, where the latest visits of all components must be inside the window. Here, a component would be allowed to be updated asynchronously as long as its visiting time of any two nodes does not differ by more than *k*. The introduction of such a window would limit the search space and decrease the running times for the asynchronous search.

The tool also uses the well-known algorithm of Quine–McCluskey to minimize the Boolean functions, thus reducing the search space of possible repair operations. We also show that the minimization algorithm does not have a significant impact on the CPU time of asynchronous runs, especially with a larger number of time steps and nodes.

As future work, we propose to reduce the search space by removing symmetries when considering an asynchronous updating scheme. In other words, by choosing which nodes to visit, one can avoid testing concurrent paths reaching the same state. This could help improve the execution time for larger networks when more iterations are required.

## Additional file

**Additional file 1: Figure S1.** Visiting Sequence. One of many possible visiting sequence performed by the methodwhen considering an asynchronous updating scheme. The green (red) colour represent theassignment of a node to the value true (false). This Figure complements the content of Table3, choosing a specific visiting order.

## Abbreviations

ASP: Answer Set Programming
STG: state transition graph
DNF: disjunctive normal form

## Footnotes

1 The Herbrand universe *H* of program *P* is the set of all ground terms, which can be constructed by the constants and function symbols from this program. For more details see [31].

2 Used minimization algorithm from https://github.com/pfpacket/Quine-McCluskey.

3 The lambda parameter represents the average number of events in one interval.
