# Supplementary figures and images for: Repairing Boolean logical models from time-series data using Answer Set Programming
Source: Algorithms Mol Biol. 2019 Mar 25;14:9. doi: 10.1186/s13015-019-0145-8 (PMC6434824; doi:10.1186/s13015-019-0145-8)

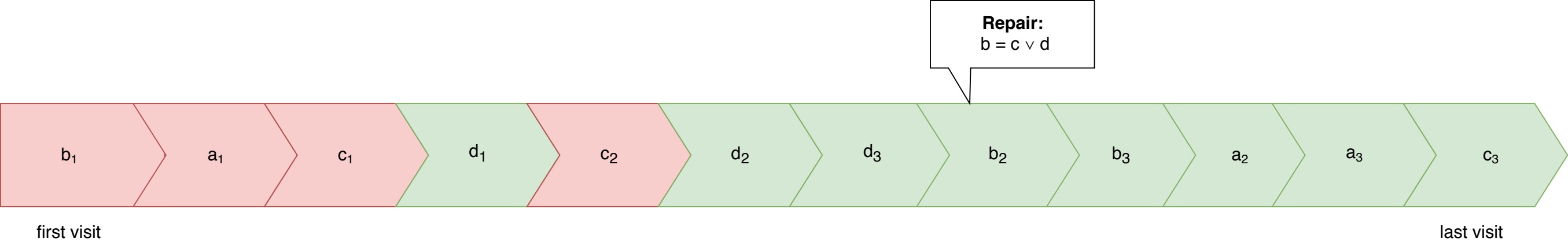

Supplement: Supplementary file 1 — Additional file 1: Figure S1. Visiting Sequence. One of many possible visiting sequence performed by the methodwhen considering an asynchronous updating scheme. The green (red) colour represent theassignment of a node to the value true (false). This Figure complements the content of Table3, choosing a specific visiting order. [file 13015_2019_145_MOESM1_ESM.pdf]
